# Poroid Hidradenoma: Dermoscopic and In Vivo Reflectance Confocal Microscopic Description

**DOI:** 10.3390/diagnostics12020255

**Published:** 2022-01-20

**Authors:** Mihai Lupu, Tiberiu Tebeica, Ana Maria Malciu, Vlad Mihai Voiculescu

**Affiliations:** 1 Department of Dermatology, “Carol Davila” University of Medicine and Pharmacy, 020021 Bucharest, Romania; voiculescuvlad@yahoo.com; 2 Department of Dermatology, Med-As Medical Centre, 030167 Bucharest, Romania; 3 Department of Dermatopathology, “Dr. Leventer Centre”, 011216 Bucharest, Romania; tiberiutebeica@drleventercentre.com; 4 Department of Dermatology and Allergology, “Elias” University Emergency Hospital, 011461 Bucharest, Romania; ana.malciu@gmail.com

**Keywords:** poroid hidradenoma, benign adnexal neoplasm, reflectance confocal microscopy, dermoscopy

## Abstract

Poroid hidradenoma (PH) is a rare, benign adnexal neoplasm usually presenting as a solitary, well circumscribed, asymptomatic papule or nodule that appears reddish and is occasionally tender. Since 1990, only a few cases of PH have been reported. We present a case of PH on the medial surface of the thigh and describe, for the first time, the dermoscopic and reflectance confocal microscopic (RCM) features in correlation with histology. A 67-year-old woman with unremarkable family or past medical history presented with a nodular lesion on the medial surface of the right thigh. The lesion had appeared 4 months earlier and rapidly enlarged. Physical examination revealed a 7 × 5 mm, non-tender, reddish nodule with clinically distinct margins. Dermoscopy showed central blue-grey pigmented areas, a polymorphous vascular pattern with arborizing, glomerular and hairpin vessels surrounded by white halos. RCM revealed an ovoid, well-outlined tumor, with a central area containing cells with distinctive morphologies, two types of tumor cells, tubular hypo-reflective structures, and rectilinear vessels in the stroma. These findings correlated with histological features, which established the diagnosis of PH. Even though the diagnosis of PH remains histopathological, non-invasive tools, such as RCM, can help rule out several malignancies, therefore reducing surgical-associated comorbidity.

## 1. Introduction

Poroid hidradenoma (PH), a benign neoplasm with eccrine differentiation, was originally described by Abenoza and Ackerman in 1990 [1]. Since then, only a few cases have been reported in the literature [2]. PH commonly presents as a solitary, well circumscribed, slightly reddish, asymptomatic lesion, with a diameter between 1–2 cm, frequently located in the head and neck area. Although the tumor has been identified in patients aged from 13 to 97 years, its peak incidence is in the sixth decade [3,4]. Histologically, PH is wholly intra-dermal, only rarely merging with the epidermis, and shows architectural features of hidradenoma and cytologic features of poroid neoplasms. 

Although PH rarely becomes malignant, the treatment consists of the complete surgical excision of the lesion in order to prevent recurrence [5]. Although PH has been histologically well defined, its dermoscopic features are rarely reported and there is no available confocal description of this tumor. In this paper, we report a case of PH with dermoscopic and reflectance confocal microscopy (RCM) findings, in correlation with histology. 

## 2. Case Report

A 67-year-old woman with unremarkable family or past medical history presented with a nodular lesion on the medial surface of her right thigh. Written informed consent was obtained and the patient agreed to the proposed diagnostic and therapeutic procedures. The study was conducted in accordance with the Declaration of Helsinki, and the protocol was approved by the Ethics Committee of the “Carol Davila” University of Medicine and Pharmacy Bucharest (Project Number 185/26.12.2018). 

**Figure 1:**
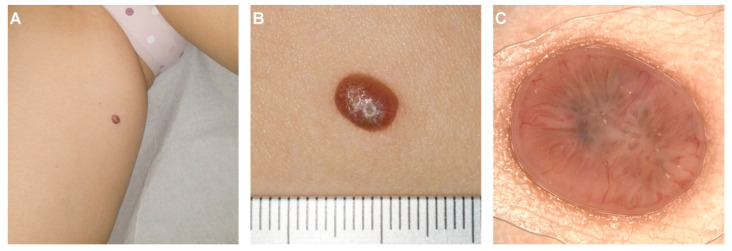
Clinical and dermoscopical aspect of the lesion. (**A**) Solitary, reddish tumor located on the medial aspect of the right thigh of a 67-year-old woman. (**B**) Close-up image of the tumor in panel (**A**) (measuring scale graded in centimeters)—central umbilication and slight desquamation on the lesion surface are noticeable. (**C**) Dermoscopically, the lesion was typified by the presence of a central blue-grey pigmented area, a peripheric polymorphous vascular pattern with arborizing, glomerular and hairpin vessels surrounded by whitish halos.

Family history and past medical history were unremarkable. The lesion had appeared 4 months earlier and rapidly enlarged over this period (Figure 1A). Physical examination revealed a 7 × 5 mm, non-tender, reddish tumor with clinically distinct margins (Figure 1B). The differential diagnosis included basal cell carcinoma, an adnexal tumor (e.g., eccrine poroma, hidroacanthoma simplex, and dermal duct tumor), pyogenic granuloma, malignant eccrine poroma [6], and melanoma.

Dermoscopy showed central blue-grey pigmented areas, a peripheric polymorphous vascular pattern with arborizing, glomerular and hairpin vessels surrounded by whitish halos (Figure 1C).

**Figure 2:**
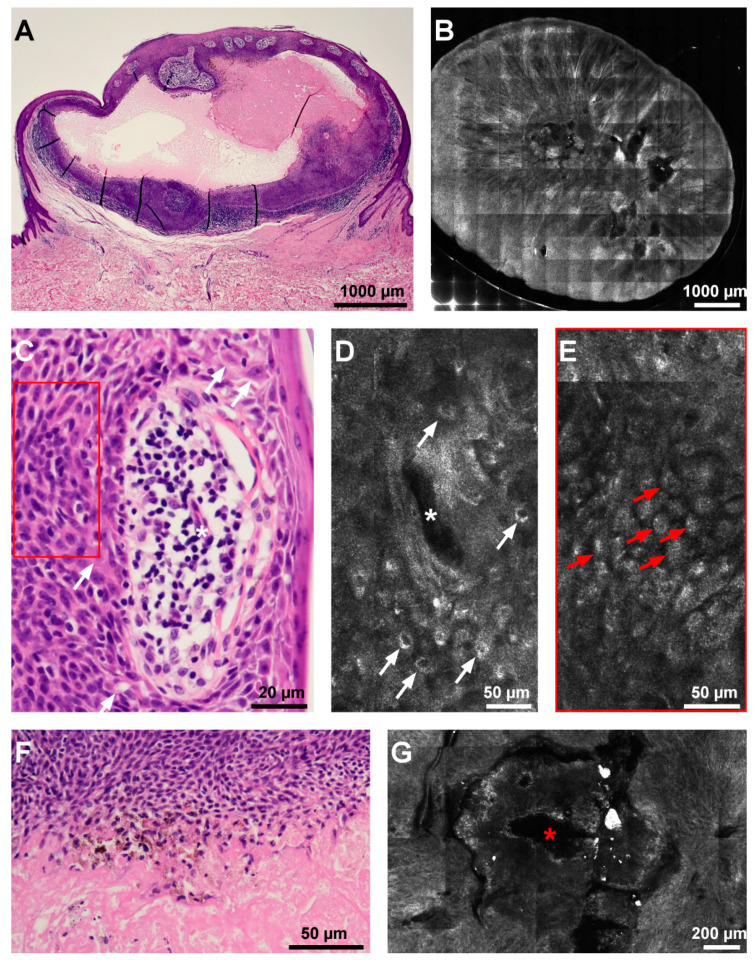
Histopathological and reflectance confocal microscopy (RCM) aspects of the lesion. (**A**) Histological architecture of the tumor at scanning magnification shows an intra-dermal, nodular proliferation containing with central cystic transformation (hematoxylin–eosin). (**B**) RCM mosaic depicts a solid tumor with a central cystic space filled with amorphous, acellular material, and linear blood vessels radiating from the lesion center. (**C**) Histopathology photomicrograph (hematoxylin–eosin) showing cuticular cells (white arrows) showing pale nuclei around a duct (white asterisk) corresponding on RCM (**D**) to round, hyporeflective cells with bright contours (white arrows) around a canalicular hyporeflective space (white asterisk); poroid cells are towards the periphery of the image (red rectangle). (**E**) RCM image displaying round, medium-reflective cells with small nuclei (red arrows) corresponding to poroid cells ((**C**) red rectangle). (**F**) Necrotic cells at the edge of a cystic space representing necrotic cells (hematoxylin–eosin). (**G**) RCM mosaic showing an area of amorphous material with medium reflectivity (red asterisk), corresponding to the area of necrosis en masse in panel (**F**).

The RCM examination revealed an ovoid, well-outlined tumor, with a central area containing amorphous material with medium reflectivity and cells with bizarre morphologies (Figure 2G). Two types of tumor cells could be identified: large, round cells with hyper-reflective contours between which there were tubular hypo-reflective spaces (Figure 2D), and more abundant, smaller, round, moderately reflective cells (Figure 2E). The stroma contained rectilinear vessels, radiating from the center of the lesion (Figure 2B). 

The lesion was surgically excised under local anesthesia. Histological examination on haematoxylin-and-eosin-stained paraffin sections revealed round, pale cells with large nuclei and clear cytoplasm (cuticular cells), small, round cells with uniform nuclei and scant basophilic cytoplasm (poroid cells) (Figure 2C), and areas of squamous metaplasia. The arrangement of cells at the periphery of vessels, cystic changes containing innumerable necrotic cells (necrosis en masse) and amorphous eosinophilic material (Figure 2F), and plasma cell infiltration were also present. The histological aspect was suggestive of a poroid hidradenoma (Figure 2A).

The patient received no further therapy, and no signs of recurrence were noticed 12 months after surgery.

## 3. Discussion

Poroid hidradenoma is an unusual form of the eccrine poroma, a hybrid lesion displaying features of both poroma and hidradenoma [7]. PH seems reddish, but the presence of cystic spaces may give it a blue tint, due to the Tyndall phenomenon [6], observable in our case during dermoscopic examination. The most commonly involved sites are the head and neck region, with a predilection for the centro-facial area. Less frequent sites include the axillae, trunk and extremities [8]. Aside from the uncommon location, on the medial surface of the thigh, our case fits this general description.

Although clinically similar to a wide range of cutaneous tumors, the presented lesion differs in its dermoscopic and confocal aspect.

Dermoscopically, PH did not present with thick, in focus, arborizing vessels, as in nodular basal cell carcinoma [9]. The white-blue color was not present at the periphery, but rather in the center of the lesion and, although polymorphous vessels were present, there were no crystalline structures and multiple erosions, as in eccrine poroma [10]. In our case, the lesion did not show whitish globular structures surrounded by homogenous, pigmented lines as in hidroacanthoma simplex [11]. Multiple red lagoons intersected by white lines were absent, thus also differentiating it from pyogenic granuloma [12]. The most common dermoscopic finding described in eccrine porocarcinoma is a polymorphous vascular pattern, consisting of hairpin, linear irregular, and dot vessels [13]. However, focally distributed whitish-pink areas surrounded by pinkish-white halos, were absent in our case. The absence of dermoscopic features typical for cutaneous melanoma and amelanotic melanoma [14] steered the diagnosis away from melanoma. 

Upon RCM examination, the absence of the characteristic tumor islands with peripheral palisading and clefting was noted, thus excluding nodular basal cell carcinoma [15]. Based on previous RCM descriptions, eccrine poroma presents with dark homogeneous islands, composed of small, monomorphic tumor cells [10], which were absent in this lesion. In our case, RCM did not show the bright collarette and the round and ovoid dark areas with multiple canalicular structures and blood flow, described in pyogenic granuloma [12]. The RCM description of eccrine porocarcinoma as epidermal, round refractile tumoral islands containing atypical, nonpalisading, small, cuboidal cells with hypo-reflective nuclei and hyper-reflective cytoplasm, and dark areas of ductal differentiation [16] did not fit the characteristics of the presented tumor. Furthermore, no criteria of a melanocytic proliferation were detected upon RCM and thus a diagnosis of melanoma was excluded.

Histopathologically, in poroid hidradenoma, neoplastic poroid cells are typically located within the dermis, the proliferation rarely being connected to the epidermis. The tumor shows architectural traits of hidradenoma, with both solid and cystic areas and cytological features of poroid neoplasms, such as poroid and cuticular cells [7]. In our case, the constellation of histological features clearly led to the diagnosis of PH, with the peculiarity of the tumor’s connection with the epidermis. In fact, the epidermal connection was clinically observable as the slight central umbilication of the lesion. Even though the diagnosis of PH is histopathological still, there are reports on the value of fine needle aspiration cytology in the diagnosis of cutaneous lesions with cystic changes [17].

Lately, imaging techniques, such as ex vivo RCM, have been gaining ground in dermatological surgery, due to their ease of use and the short delay to diagnosis [18]. Concerning rare skin tumors, such as poroid hidradenoma, ex vivo RCM has been successfully used in the precise characterization and diagnosis of an atypical fibroxanthoma [19], thus allowing for a proper therapeutic course.

In our case, the in vivo RCM examination showed a central area containing amorphous material and cells with bizarre morphologies correlated histologically to necrosis en masse, two types of tumor cell morphologies (poroid and cuticular), and duct formation (tubular hypo-reflective spaces), features which correlated well with histology findings.

Poroid hidradenoma is managed by surgical excision in order to prevent its recurrence. The prognosis is generally very good, although recurrences have been reported in a few cases [17].

## 4. Conclusions

Poroid hidradenoma is the newest member in the poroma group. Although it rarely becomes malignant (less than 1% of cases) [5], it can easily be mistaken for a malignant lesion. While the confocal features of eccrine poroma have already been described [10], to the best of our knowledge, this is the first reported RCM description of PH. In such cases, non-invasive tools, such as dermoscopy and RCM, can help to rule out melanoma and non-melanoma skin cancers. The small number of reported cases has led to a limited understanding of this entity. As such, PH should remain within the differential diagnosis of practitioners.

## Data Availability

The data presented in this study are available on request from the corresponding author.
